# Efficacy of azithromycin combined with compounded atovaquone in treating babesiosis in giant pandas

**DOI:** 10.1186/s13071-024-06615-9

**Published:** 2024-12-23

**Authors:** Rui Ma, Chanjuan Yue, Jiang Gu, Wei Wu, Rong Hou, Wenjun Huang, Bi Li, Fei Xue, Chong Huang, Wenlei Bi, Jiabin Liu, Xiang Yu, Zusheng Li, Wanjing Yang, Mingxia Fu, Hong Yang, Dunwu Qi

**Affiliations:** 1 Sichuan Key Laboratory of Conservation Biology for Endangered Wildlife, Chengdu Research Base of Giant Panda Breeding, Chengdu, 610000 Sichuan China; 2 Administration of Daxiangling Nature Reserve, Yaan, 625000 Sichuan China

**Keywords:** Clinical efficacy, Azithromycin, Atovaquone, Compounded treatment, *Babesia*, Giant panda

## Abstract

**Background:**

Babesia is a tick-borne protozoan blood parasite that can cause hemolytic anemia, thrombocytopenia, lethargy and splenomegaly in giant pandas.

**Methods:**

We evaluated the efficacy and safety profile of a therapeutic regimen combining atovaquone and zithromycin in the context of babesiosis in giant pandas that have been naturally infected. The examined pandas underwent clinical and laboratory analyses, including hematology, biochemistry and thyroid hormone profiles. Upon diagnosis, the giant pandas were administered a compounded treatment consisting of atovaquone oral suspension (15 mg/kg, PO, q8 h), azithromycin tablets (10 mg/kg, PO, q24 h) and Enteral Nutritional Suspension (TPF) as a fat-rich supplement (0.5 ml/kg, PO, q8 h) for a 10-day period.

**Results:**

The combination treatment increased the red blood cell count, hemoglobin levels and hematocrit in the pandas within a short period, while also reducing parasite levels below the PCR detection threshold.

**Conclusions:**

Our study suggested that atovaquone and azithromycin combination therapy is highly effective for emergency treatment of *Babesia* sp. infection in giant pandas.

**Graphical Abstract:**

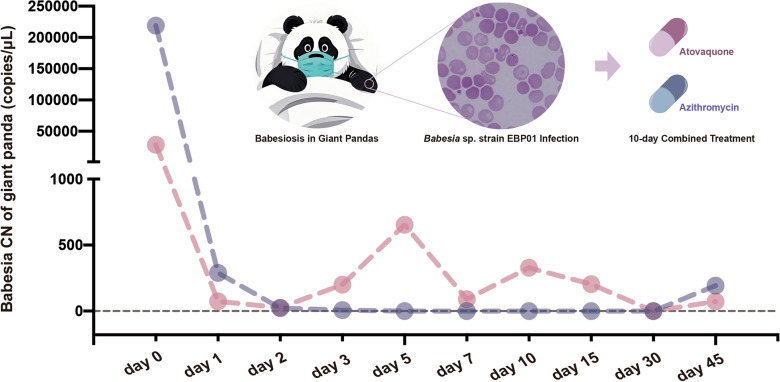

**Supplementary Information:**

The online version contains supplementary material available at 10.1186/s13071-024-06615-9.

## Background

Babesiosis is a globally distributed tick-borne disease caused by intraerythrocytic protozoa of the genus *Babesia* [1]. It is widely prevalent in tropical, subtropical and temperate climates and represents a major health concern worldwide, affecting both human and animal populations [1–3]. Babesiosis manifests in a spectrum from mild, asymptomatic infections to severe cases involving multiple organ failure and, in extreme cases, death [1, 3]. Giant pandas are also threatened by babesiosis. In our previous study, *Babesia* sp*.* infection was detected in giant pandas, now referred to as *Babesia* sp. strain EBP01, which may represent a novel species [4]. The individual giant panda infected with *Babesia* sp. strain EBP01 exhibited symptoms such as fever, anemia, jaundice and hemoglobinuria [4].

The clinical signs of babesiosis include hemolytic anemia, fever, jaundice and hemoglobinuria, and some serious cases result in death [5]. The therapeutic landscape for *Babesia* sp. infection incorporates a regimen of imidocarb dipropionate [6], diminazene aceturate [7], atovaquone [8] and a suite of antibiotics such as doxycycline [9], clindamycin [10], enrofloxacin [11] and azithromycin [12]. A synergistic approach, combining specific drugs with antibiotics, generally results in more favorable outcomes and lower rates of recurrence compared to using a single drug [13, 14]. Among these combinations, using atovaquone paired with azithromycin is particularly advocated, despite the associated higher costs [15]. Recent research has highlighted the efficacy of a novel combination treatment involving atovaquone-proguanil (Malarone^®^), azithromycin (AZM) and the herbal derivative artesunate (ART) in managing infections caused by *Babesia gibsoni* in canines; this regimen has been shown to sustain remission for an impressive duration of 720 days [16]. Notably, however, the effectiveness of this combination has yet to be confirmed for other animal species or different strains of *Babesia* spp. Thus, a need remains for an effective therapeutic agent for the treatment of giant panda babesiosis caused by *Babesia* sp. [4].

Currently, no evidence has been published on the therapeutic activity of atovaquone combined with azithromycin against *Babesia* sp. strain EBP01 of giant panda. This study examines the effectiveness, safety and recurrence rates of combination therapy in treating giant pandas naturally infected with *Babesia* sp. The results offer promising evidence for developing preventive measures and treatments for babesiosis in the endangered animals and also provide crucial insights that could guide the management of similar infections in other endangered wildlife species globally.

## Methods

### Subjects

Two giant pandas naturally infected with *Babesia* sp. were found at the Chengdu Research Base of Giant Panda Breeding through the parasite disease survey, confirmed using PCR assay. In the previous study [4], detailed descriptions of the morphological and molecular biological results were given, so they will not be repeated in this article. The two giant pandas did not exhibit any marked clinical signs of *Babesia* sp. infection, including symptoms such as lethargy, weight loss, reduced activity or listlessness. Regular monthly medical check-ups consistently showed no evidence of susceptibility to common infectious diseases like canine distemper virus (CDV), canine parvovirus (CPV), feline panleukopenia virus (FPV), canine rotavirus (CRV) and rabies virus (RV). The main criterion for inclusion of the samples in the study was *Babesia* real-time fluorescent quantitative PCR (qPCR) positivity. Two pandas in our study had not received any treatment with antibiotics, antifungals, corticosteroids and/or a specific anti-*Babesia* agent within 360 days prior to PCR confirmation of *Babesia* sp.

This study involved two 5-year-old female giant pandas weighing 85 kg and 100 kg, respectively. The subjects underwent clinical and laboratory examinations, including hematological and biochemical analyses and qPCR testing. Post diagnosis and initial clinical evaluations, the pandas received treatment with atovaquone and azithromycin, and subsequent monitoring was conducted to gauge changes in clinical indicators. Over the course of the study, hematological, biochemical and qPCR analyses were performed on days 0 (prior to treatment initiation), 1, 2, 3, 5, 7, 10, 15, 30 and 45 to assess the effectiveness of the therapy in giant pandas.

### Evaluation of clinical parameters

All giant pandas underwent comprehensive blood hematological, biochemical and thyroid hormone analyses and qPCR detection before treatment (day 0) and after treatment (days 1, 2, 3, 5, 7, 10, 15, 30, 45). Each giant panda individual was well trained for blood collection and completed the procedure without anesthesia. Blood collection was conducted in the enclosure using disposable sterile needles for venipuncture. Each panda contributed three types of blood samples. A 2-ml blood sample was placed in an EDTA tube and evenly divided for hematological analysis and quantitative detection of *Babesia* by qPCR, respectively: 1 ml was collected in a heparin sodium anticoagulant tube for biochemical analysis; 1 ml sample of non-anticoagulated blood was centrifuged for 15 min at 253 g (F-20/micro, Thermo Fisher Scientific, Waltham, MA, USA) to evaluate the impact of the treatment on thyroid function by serum direct chemiluminescence technology. This rigorous approach not only maintained the well-being of the animals but also ensured the integrity and reliability of the collected data. Complete hematological analysis was done using the ProCyte Dx automated hematology analyzer (IDEXX Laboratories, Westbrook, ME, USA). Blood biochemical parameters were measured using the FUJI automatic dry chemistry analyzer (DRI-CHEM NX500iVC, FUJIFILM, Tokyo, Japan). Free triiodothyronine (fT3), free thyroxine (fT4), triiodothyronine (T3) and thyroxine (T4) were measured using the direct chemiluminescence method by assay kits (ADVIA^®^Centaur, SIEMENS Healthineers, Inc.) according to the manufacturer’s instructions.

### DNA extraction

DNA was extracted from 200 μl of whole blood using the QIAamp 96 DNA Blood Kit (Qiagen, Hilden, Germany) and eluted in 100 μl of elution buffer. Following the manufacturer’s instructions, the DNA concentration was measured with a NanoDrop 2000 spectrophotometer (Thermo Fisher Scientific, Wilmington, DE, USA). The extracted DNA was stored at – 20 ℃ for subsequent analysis.

### Quantitative PCR detection of *Babesia* sp.

*Babesia* was detected using SYBR Green qPCR using chromosomal DNA as a template. The CFX96 Real-Time PCR System (Bio-Rad, USA) was utilized to perform qPCR, employing the primers F1 (5′-GGG AGG TAG TGA CAA GAA ATA A-3′) and R1 (5′-TACG AAG TCG ATA ACG CAG A-3′). The primers were self-designed based on the EPB01 18S rRNA sequence and verified by sequencing, which amplify a 197-bp fragment. The optimized qPCR reaction was prepared in a total volume of 10 μl containing 5 μl of iTaq Universal SYBR Green PCR Supermix (2 ×) (Bio-Rad, USA), 0.5 μl of each primer (F1 and R1, 4 μM), 3 μl ultrapure water and 1 μl DNA template. The qPCRs reactions were conducted in duplicate. The thermocycling conditions were as follows: an initial denaturation at 95 ℃ for 5 min, followed by 40 cycles at 95 ℃ for 10 s and 56 ℃ for 30 s, and a subsequent dissociation curve analysis. A plasmid containing the EPB01 18S rRNA sequence was used as a positive control. To prevent contamination, barrier pipette tips were used, and a negative control was included in each set of runs by adding 1 μl ultrapure water. To estimate the quantity of DNA, a standard curve was generated from amplification products of *Babesia* sp. 18S rRNA plasmids.

### Treatment protocol and monitoring

The treatment regimen included administering azithromycin tablets (10 mg/kg, PO, q24 h; Zithromax, Pfizer) and atovaquone oral suspension (15 mg/kg, PO, q8 h; Wellvone, GlaxoSmithKline) with a fatty meal; they were compounded into capsules for a 10-day course. Given the giant panda’s unique herbivorous diet, Enteral Nutritional Suspension (TPF; Nutrison Fibre, Nutricia) was selected to complement fatty meals (0.5 ml/kg, PO, q8 h). The treatment response was monitored via qPCR testing for *Babesia* sp. on Days 1, 2, 3, 5, 7, 10, 15, 30 and 45. Pandas that tested positive for *Babesia* sp. at any point during these intervals underwent retesting at the 30-day mark. A successful outcome was defined as two consecutive negative PCR results spaced 30 days apart.

### Statistical analysis

For statistical analyses, a GraphPad Prism 8.0 (GraphPad, San Diego, CA, USA) software package was used. One-way ANOVA test was used to perform pairwise comparisons for data sets obtained between blood tests before treatment (Day 0) and each datum during the treatment (Days 1–10) and monitoring period (Day 11–45). A simple linear regression model was used to identify significantly affected hematological, biochemical and thyroid hormone parameters, and the results were displayed using scatter plots. The hematological, biochemical and thyroid hormone parameter values are expressed as the analyzed specific numerical values. The quantitative PCR results for *Babesia* sp. in two individual samples were presented using a line graph. Statistical significance level was set to *p* < 0.05.

## Results

### Clinical efficacy

On the first day (Day 1) of the combined therapy, one subject experienced mild discomfort, manifested as lethargy, reduced food intake and curling up, which lasted for 2 h (1/2; 50.0%). Subsequently, the two subjects’ symptoms were alleviated, and they returned to a normal state, resuming feeding and displaying typical behavior. Furthermore, no adverse reactions occurred throughout the remainder of the treatment period (continuing until day 10). By day 45, no clinical symptoms such as pale mucous membranes, exercise fatigue or fever were observed, excluding periodic intestinal mucous secretion and excretion specific to giant pandas. During the treatment and monitoring period, no significant adverse reactions were reported.

### Clinicopathological abnormalities

**Fig. 1:**
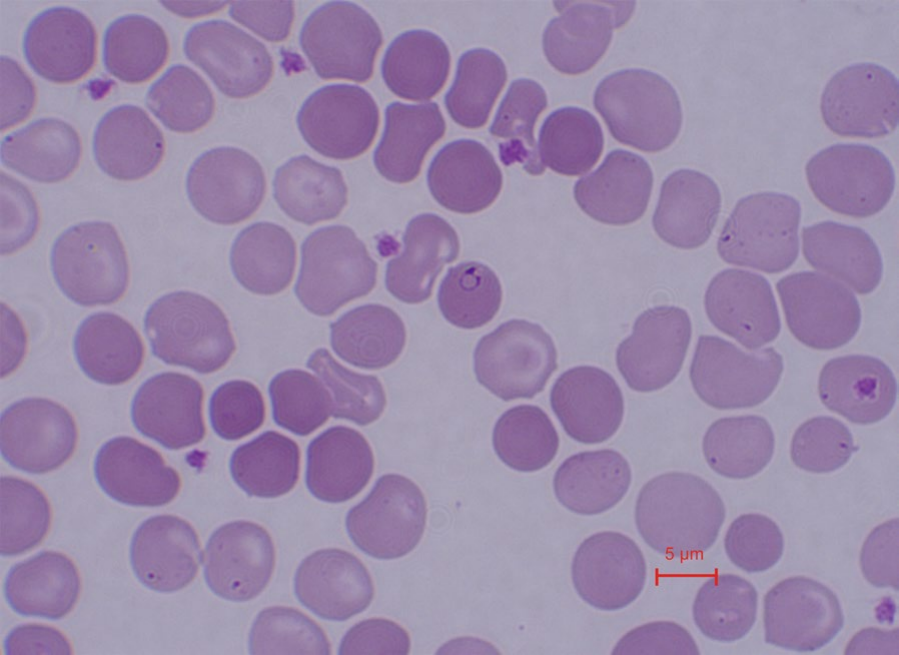
Giemsa-stained thin blood smear of *Babesia* sp. from the giant panda. Final magnification of 1000×

**Fig. 2:**
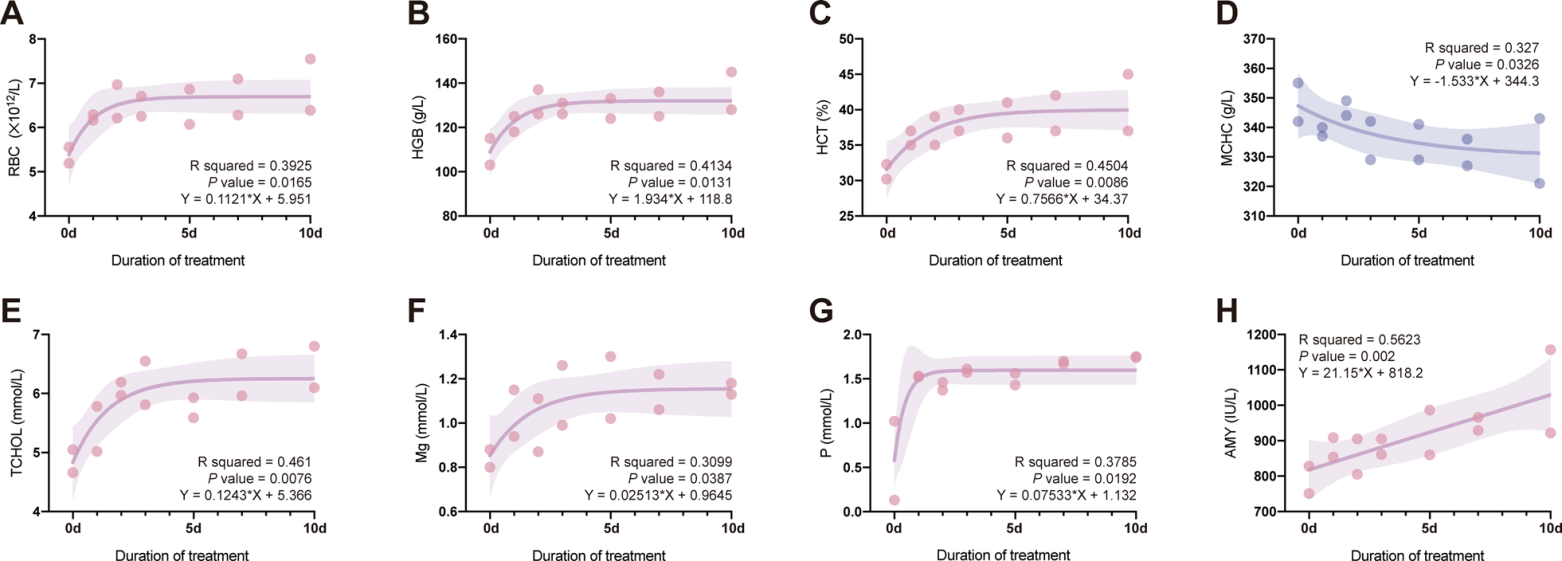
Correlation results between combined treatment and blood test parameter indicators. Correlation results from the treatment process with: **A** red blood cells (RBC); **B** hemoglobin (HGB); **C** hematocrit (HCT); **D** mean corpuscular hemoglobin (MCHC); **E** total cholesterol (TCHOL); **F** magnesium (Mg); **G** phosphorus (P) and **H** blood amylase (AMY). The x-axis represents the treatment duration (cumulative drug intake), and the y-axis represents the specific values of these indicators. Red indicates a significant positive correlation, while blue indicates a significant negative correlation

The Diff Quik staining results of the blood smear of the giant panda show that the parasite is round with a 1.74–3.05 μm × 0.89–1.54 μm diameter, *n* = 14 (Fig. 1). Tables 1 and 2 summarize the laboratory parameters of the two giant pandas during the treatment period (Day 1 to Day 10) and the monitoring period (Day 11 to Day 45). The laboratory analysis revealed anemia in 2/2 giant pandas before treatment (HGB: 103 g/l and 115 g/l, respectively; HCT: 32.2% and 30.2%, respectively). Laboratory analyses indicated that the red blood cell count (RBC, 4.72–8.04 × 10^12^/l), hemoglobin concentration (HGB, 118.00–167.00 g/l) and hematocrit (HCT, 35.4–44.3%) increased after the first day of treatment and remained within the normal range for over 45 days. Although the differential analysis revealed no significant differences before and after treatment (all *p* > 0.05, Table S1), further univariate linear regression analysis demonstrated a significant positive correlation between the duration of combined treatment (cumulative drug dosage) and increases in RBC (*R*^2^ = 0.3925, *p* = 0.0165, Fig. 2A), HGB (*R*^2^ = 0.4134, *p* = 0.0131, Fig. 2B) and HCT (*R*^2^ = 0.4504, *p* = 0.0086, Fig. 2C). Additionally, we observed that the mean corpuscular hemoglobin concentration (MCHC) in individuals’ blood exhibited a significant negative correlation with the duration of the treatment period (*R*^2^ = 0.3270, *p* = 0.0326, Fig. 2D) and returned to pre-treatment levels upon completion of the 10-day treatment. The findings suggest that the combined use of atovaquone and azithromycin significantly ameliorates the symptoms associated with *Babesia* sp. infection, including reductions in RBC, HGB and HCT.

**Table 1:** Hematological, biochemical and thyroid hormone parameters in giant panda 1 showing clinical remission for babesiosis following treatment with atovaquone and azithromycin combination

Blood test	Parameter	Reference range	Day0	Day1	Day2	Day3	Day5	Day7	Day10	Day15	Day30	DAY45
Hematological test	WBC (×10^9^/l)	4.20–11.10	7.24	7.63	7.48	6.88	7.05	6.79	7.25	7.29	7.41	8.14
	LYM (%)	24.0–49.0	31.6	22.8	36.9	33.3	35.3	39.3	36.1	41.4	39.5	25.3
	MON (%)	< 7.0	10.4	12.3	6.2	7.8	6.9	5.7	8.8	4.9	5.3	8.6
	EOS (%)	0.0	0.0	2.3	0.0	0.0	0.0	0.0	0.1	0.0	0.0	0.0
	BAS (%)	< 0.4	0.1	0.0	0.0	0.1	0.0	0.9	0.3	0.0	0.1	0.1
	NEU (%)	66.1–79.4	57.9	62.6	56.9	58.8	57.8	54.1	54.7	53.6	55.1	66.0
	RBC (×10^12^/l)	4.72–8.04	5.19	6.29	6.97	6.71	6.86	7.10	7.55	6.96	7.08	6.81
	HGB (g/l)	118.00–167.00	103.00	118.00	137.00	131.00	133.00	136.00	145.00	133.00	137.00	137.00
	HCT (%)	35.4–44.3	30.2	35.0	39.0	40.0	41.0	42.0	45.0	40.0	41.0	39.4
	MCV (fl)	53.10–59.70	58.20	55.60	56.30	59.50	59.10	58.50	59.90	57.70	58.00	57.90
	MCH (pg)	19.20–21.70	19.90	18.80	19.70	19.50	19.50	19.10	19.20	19.20	19.30	20.10
	MCHC (g/l)	355.00–393.00	342.00	337.00	349.00	329.00	329.00	327.00	321.00	332.00	333.00	348.00
	RDW-CV (%)	15.0–17.0	17.6	15.3	15.8	16.0	16.0	15.9	16.3	15.9	16.0	18.0
	RDW-SD (%)	31.7–34.1	37.2	29.0	32.0	34.4	34.1	33.7	35.3	33.2	33.5	36.6
	PLT (×10^9^/l)	320.00–521.00	383.00	578.00	568.00	609.00	626.00	556.00	561.00	651.00	623.00	412.00
	MPV (fl)	5.90–6.40	6.30	7.20	6.00	5.90	5.80	5.50	5.90	5.80	5.50	5.70
	PCT (%)	0.3–0.4	5.6	5.5	4.7	3.9	3.9	1.9	4.1	3.2	3.2	4.0
Biochemical test	ALT (IU/l)	25.00–66.42	100.00	86.00	85.00	87.00	87.00	90.00	85.00	88.00	91.00	91.00
	AST (IU/l)	44.00–214.00	76.00	57.00	45.00	50.00	67.00	63.00	67.00	58.00	56.00	57.00
	AKP (IU/l)	93.00–149.60	198.00	101.00	93.00	103.00	103.00	105.00	93.00	103.00	102.00	106.00
	TP (g/l)	61.60–75.20	71.70	74.90	75.90	72.90	73.10	75.50	74.60	71.00	72.60	72.80
	ALB (g/l)	34.10–39.00	34.90	38.00	38.90	40.20	39.90	39.20	39.80	39.40	40.30	40.70
	GLB (g/l)	27.5–39.40	36.80	36.90	37.00	32.70	33.20	36.30	34.80	31.60	32.30	32.10
	TBIL (μmol/l)	5.07–6.14	1.01	1.20	1.20	0.80	1.10	0.90	1.00	1.10	0.90	1.50
	DBIL (μmol/l)	0.70–4.00	0.21	1.00	0.20	0.40	0.30	0.70	0.20	1.10	0.80	0.50
	IBIL (μmol/l)	0.29–0.89	0.80	0.20	0.90	0.40	0.80	0.20	0.80	0.00	0.10	1.00
	GLU (mmol/l)	3.38–6.10	4.76	4.97	8.72	6.48	7.04	8.07	6.60	8.19	7.07	6.78
	BUN (mmol/l)	4.10–6.21	6.56	3.38	3.45	5.70	3.73	3.70	3.94	2.83	3.18	3.61
	CREA (μmol/l)	82.2–132.5	60.00	158.00	169.00	168.00	160.00	168.00	171.00	158.00	169.00	175.00
	TRIG (mmol/l)	1.00–1.90	1.27	7.00	7.11	7.15	6.55	6.85	6.53	6.10	6.15	6.25
	TCHOL (mmol/l)	6.12–10.56	4.66	5.78	6.19	6.55	5.93	6.67	6.80	6.42	6.95	6.58
	HDL-C (mmol/l)	3.33–4.10	2.59	2.22	2.31	2.49	2.35	2.46	2.46	2.42	2.55	2.45
	LDL-C (mmol/l)	0.54–3.07	0.67	1.16	1.34	1.32	1.72	1.46	1.69	1.00	1.04	1.09
	LDH (IU/l)	248.00–917.00	1539.90	1268.00	1026.00	997.00	1023.00	1300.00	1208.00	1189.00	1157.00	1265.00
	Ca (mmol/l)	2.40–2.60	2.07	2.50	2.51	2.56	2.44	2.62	2.48	2.45	2.69	2.58
	Mg (mmol/l)	0.83–1.10	0.80	0.94	0.87	0.99	1.02	1.06	1.18	1.00	1.08	0.98
	P (mmol/l)	1.10–1,70	0.13	1.52	1.46	1.61	1.56	1.67	1.75	1.79	1.83	1.85
	CO2-cp (mmol/l)	16.47–21.29	15.00	11.00	13.00	12.00	12.00	13.00	14.00	13.00	13.00	13.00
	AMY (IU/l)	149.40–617.40	751.00	854.00	805.00	861.00	860.00	929.00	922.00	938.00	987.00	988.00
Thyroid hormone test	T3 (nmol/l)	0.22–0.88	0.25	0.20	0.20	0.20	0.20	0.20	0.20	0.20	0.20	0.20
	T4 (nmol/l)	2.80–4.40	3.90	0.50	0.50	0.50	0.50	0.50	0.50	0.50	0.50	0.50
	fT3 (pmol/l)	2.50–3.30	2.49	1.00	1.05	0.93	1.05	1.03	0.97	0.93	0.96	1.10
	fT4 (pmol/l)	6.20–7.60	7.31	5.00	5.10	4.40	4.40	4.00	4.20	4.10	4.00	4.40

Data are expressed as mean ± SD

*WBC* white blood cell, *LYM* lymphocyte, *MON* monocyte, *EOS* eosinophil, *BAS* basophilic granulocyte, *NEU* neutrophilic granulocyte, *RBC* red blood cells, *HGB* hemoglobin, *HCT* hematocrit, *MCV* mean corpuscular hemoglobin concentration, *MCH* mean corpuscular hemoglobin, *MCHC* mean corpuscular hemoglobin concentration, *RDW-CV* red blood cell volume distribution width-coefficient of variation, *RDW-SD* red blood cell volume distribution width-standard deviation, *PLT* platelet counts, *MPV* mean platelet volume, *PCT* procalcitonin, *ALT* alanine amino transferase, *AST* aspartate aminotransferase; *AKP* alkaline phosphatase, *TP* total protein, *ALB* albumin, *GLB* globulin, *TBIL* total bilirubin, *DBIL* direct bilirubin, *IBIL* indirect bilirubin, *GLU* glucose, *BUN* blood urea nitrogen, *CREA* creatinine, *TRIG* triglyceride, *TCHOL* total cholesterol, *HDL-C* high-density lipoprotein-cholesterol, *LDL-C* low-density lipoprotein cholesterol, *LDH* lactic dehydrogenase, *Ca* calcium, *Mg* magnesium, *P* phosphate, *CO*_*2*_ carbon dioxide binding rate, *AMY* serum amylase, *T3* triiodothyronine, *T4* tetraiodothyronine, *fT3* free triiodothyronine, *fT4* free tetraiodothyronine

**Table 2:** Hematological, biochemical and thyroid hormone parameters in giant panda 2 showing clinical remission for babesiosis following treatment with atovaquone and azithromycin combination

Blood test	Parameter	Reference range	Day0	Day1	Day2	Day3	Day5	Day7	Day10	Day15	Day30	Day45
Hematological test	WBC (× 10^9^/l)	4.20–11.10	7.01	7.01	7.01	7.60	7.48	8.74	8.74	6.15	7.80	7.80
	LYM (%)	24.0–49.0	19.1	19.1	19.1	18.4	16.9	15.5	15.5	24.4	16.8	27.4
	MON (%)	< 7.0	10.2	10.2	10.2	7.0	8.9	6.4	6.4	5.6	6.8	6.2
	EOS (%)	0.0	0.0	0.0	0.0	0.0	0.0	0.0	0.0	0.0	0.0	0.0
	BAS (%)	< 0.4	0.2	0.2	0.2	0.0	0.1	0.1	0.1	0.1	0.2	0.0
	NEU (%)	66.1–79.4	70.5	70.5	70.5	74.0	74.1	78.0	78.0	69.9	76.2	66.4
	RBC (×10^12^/l)	4.72–8.04	5.56	5.56	5.56	6.25	6.07	6.28	6.28	7.16	6.08	6.40
	HGB (g/l)	118.00–167.00	115.00	115.00	115.00	126.00	124.00	125.00	125.00	144.00	123.00	124.00
	HCT (%)	35.4–44.3	32.3	32.3	32.3	37.0	36.0	37.0	37.0	39.0	36.0	35.0
	MCV (fl)	53.10–59.70	58.20	58.20	58.20	58.90	59.80	59.40	59.40	55.10	59.10	53.90
	MCH (pg)	19.20–21.70	20.60	20.60	20.60	20.10	20.40	20.00	20.00	20.10	20.20	19.30
	MCHC (g/l)	355.00–393.00	355.00	355.00	355.00	342.00	341.00	336.00	336.00	365.00	342.00	358.00
	RDW-CV (%)	15.0–17.0	16.6	16.6	16.6	16.3	16.3	16.0	16.0	15.4	15.9	17.0
	RDW-SD (%)	31.7–34.1	35.0	35.0	35.0	34.6	35.1	34.3	34.3	29.5	33.9	31.7
	PLT (×10^9^/l)	320.00–521.00	470.00	470.00	470.00	502.00	553.00	554.00	554.00	340.00	584.00	802.00
	MPV (fl)	5.90–6.40	6.80	6.80	6.80	7.90	8.10	7.40	7.40	5.50	8.10	6.40
	PCT (%)	0.3–0.4	9.3	9.3	9.3	15.7	15.9	13.2	13.2	3.3	16.1	6.5
Biochemical test	ALT (IU/l)	25.00–66.42	77.00	102.00	154.00	139.00	126.65	140.00	143.00	139.00	133.00	146.00
	AST (IU/l)	44.00–214.00	96.00	54.00	103.00	63.00	88.47	55.00	64.00	60.00	62.00	82.00
	AKP (IU/l)	93.00–149.60	60.00	161.00	201.00	203.00	182.08	223.00	225.00	212.00	222.00	233.00
	TP (g/l)	61.60–75.20	75.80	70.60	70.40	66.30	74.31	68.90	67.40	67.20	66.20	65.20
	ALB (g/l)	34.10–39.00	37.70	37.40	37.10	36.00	35.56	36.40	35.80	36.60	35.10	34.10
	GLB (g/l)	27.5–39.40	38.10	33.20	33.30	30.30	38.75	32.50	31.60	30.60	31.10	31.10
	TBIL (μmol/l)	5.07–6.14	0.20	1.90	1.00	0.70	0.99	0.80	1.10	1.10	0.60	1.70
	DBIL (μmol/l)	0.70–4.00	0.08	1.10	0.60	0.30	0.61	0.00	0.20	0.10	0.20	0.90
	IBIL (μmol/l)	0.29–0.89	0.12	0.80	0.40	0.40	0.38	0.80	0.90	1.00	0.40	0.80
	GLU (mmol/l)	3.38–6.10	6.56	4.36	7.05	6.18	3.00	4.66	4.82	5.09	3.87	4.89
	BUN (mmol/l)	4.10–6.21	6.62	9.63	3.52	4.41	4.51	4.52	4.16	4.72	4.64	3.41
	CREA (μmol/l)	82.2–132.5	83.00	147.00	158.00	154.00	94.03	168.00	175.00	166.00	156.00	162.00
	TRIG (mmol/l)	1.00–1.90	3.81	1.59	1.49	1.27	1.18	1.23	1.35	1.54	1.63	1.88
	TCHOL (mmol/l)	6.12–10.56	5.05	5.02	5.97	5.81	5.59	5.96	6.10	6.10	5.98	5.93
	HDL-C (mmol/l)	3.33–4.10	2.15	2.75	3.20	3.15	3.60	3.29	3.48	3.45	3.25	3.12
	LDL-C (mmol/l)	0.54–3.07	1.12	0.85	0.74	0.86	1.55	3.29	0.73	0.75	0.69	0.68
	LDH (IU/l)	248.00–917.00	1740.90	987.00	896.00	795.00	898.00	906.00	885.00	695.00	731.00	761.00
	Ca (mmol/l)	2.40–2.60	2.35	2.54	2.62	2.57	2.48	2.60	2.59	2.54	2.36	2.46
	Mg (mmol/l)	0.83–1.10	0.88	1.15	1.11	1.26	1.30	1.22	1.13	1.12	1.14	1.12
	P (mmol/l)	1.10–1,70	1.02	1.53	1.37	1.57	1.43	1.70	1.74	1.64	1.80	1.65
	CO2-cp (mmol/l)	16.47–21.29	16.60	13.00	15.00	14.00	18.26	16.00	15.00	15.00	15.00	14.00
	AMY (IU/l)	149.40–617.40	828.00	909.00	905.00	905.00	986.58	966.00	1157.00	1183.00	1188.00	1039.00
Thyroid hormone test	T3 (nmol/l)	0.22–0.88	0.21	0.28	0.32	0.29	0.30	0.27	0.29	0.27	0.25	0.27
	T4 (nmol/l)	2.80–4.40	1.40	0.50	0.79	0.55	0.57	0.50	0.50	0.50	0.50	0.50
	fT3 (pmol/l)	2.50–3.30	2.03	1.70	2.21	1.97	2.06	1.79	1.79	1.84	1.56	1.63
	fT4 (pmol/l)	6.20–7.60	6.98	6.10	10.80	9.40	9.10	7.60	7.20	6.70	6.00	6.10

Data are expressed as mean ± SD

*WBC* white blood cell, *LYM* lymphocyte, *MON* monocyte, *EOS* eosinophil, *BAS* basophilic granulocyte, *NEU* neutrophilic granulocyte, *RBC* red blood cells, *HGB* hemoglobin, *HCT* hematocrit, *MCV* mean corpuscular hemoglobin concentration, *MCH* mean corpuscular hemoglobin, *MCHC* mean corpuscular hemoglobin concentration, *RDW-CV* red blood cell volume distribution width-coefficient of variation, *RDW-SD* red blood cell volume distribution width-standard deviation, *PLT* platelet counts, *MPV* mean platelet volume, *PCT* procalcitonin, *ALT* alanine amino transferase, *AST* aspartate aminotransferase, *AKP* alkaline phosphatase, *TP* total protein, *ALB* albumin, *GLB* globulin, *TBIL* total bilirubin, *DBIL* direct bilirubin, *IBIL* indirect bilirubin, *GLU* glucose, *BUN* blood urea nitrogen, *CREA* creatinine, *TRIG* triglyceride, *TCHOL* total cholesterol, *HDL-C* high-density lipoprotein-cholesterol, *LDL-C* low-density lipoprotein cholesterol, *LDH* lactic dehydrogenase, *Ca* calcium, *Mg* magnesium, *P* phosphate, *CO*_*2*_ carbon dioxide binding rate, *AMY* serum amylase, *T3* triiodothyronine, *T4* tetraiodothyronine, *fT3* free triiodothyronine, *fT4* free tetraiodothyronine

Biochemistry analysis revealed a mild increase in alanine aminotransferase (ALT) and a mild decrease in aspartate aminotransferase (AST) with no statistical significance and showed no significant correlation with the treatment duration. Regarding renal function metabolism, the combined treatment had no impact on blood urea nitrogen (BUN) levels but slightly increased creatinine (CREA) levels, though these changes were not statistically significant or correlated with the treatment. Although no significant differences were observed between pre-treatment (day 0) and treatment periods, the treatment period showed a significant positive correlation with total cholesterol (TCHOL, *R*^2^ = 0.4610, *p* = 0.0076, Fig. 2E), magnesium (Mg, *R*^2^ = 0.3099, *p* = 0.0387, Fig. 2F), phosphorus (*P*, *R*^2^ = 0.3785, *p* = 0.0192, Fig. 2G) and amylase (AMY, *R*^2^ = 0.5623, *p* = 0.002, Fig. 2H). The TCHOL, Mg and P levels fluctuated within the normal reference range during and after the treatment period, with occasional minor abnormalities. Monitoring during and after treatment revealed that the combined medication regimen did not impact thyroid hormone secretion, with all four indicators (T3, T4, fT3 and fT4) remaining stable.

### PCR assay treatment reduces the capacity for detecting *Babesia* sp.

**Fig. 3:**
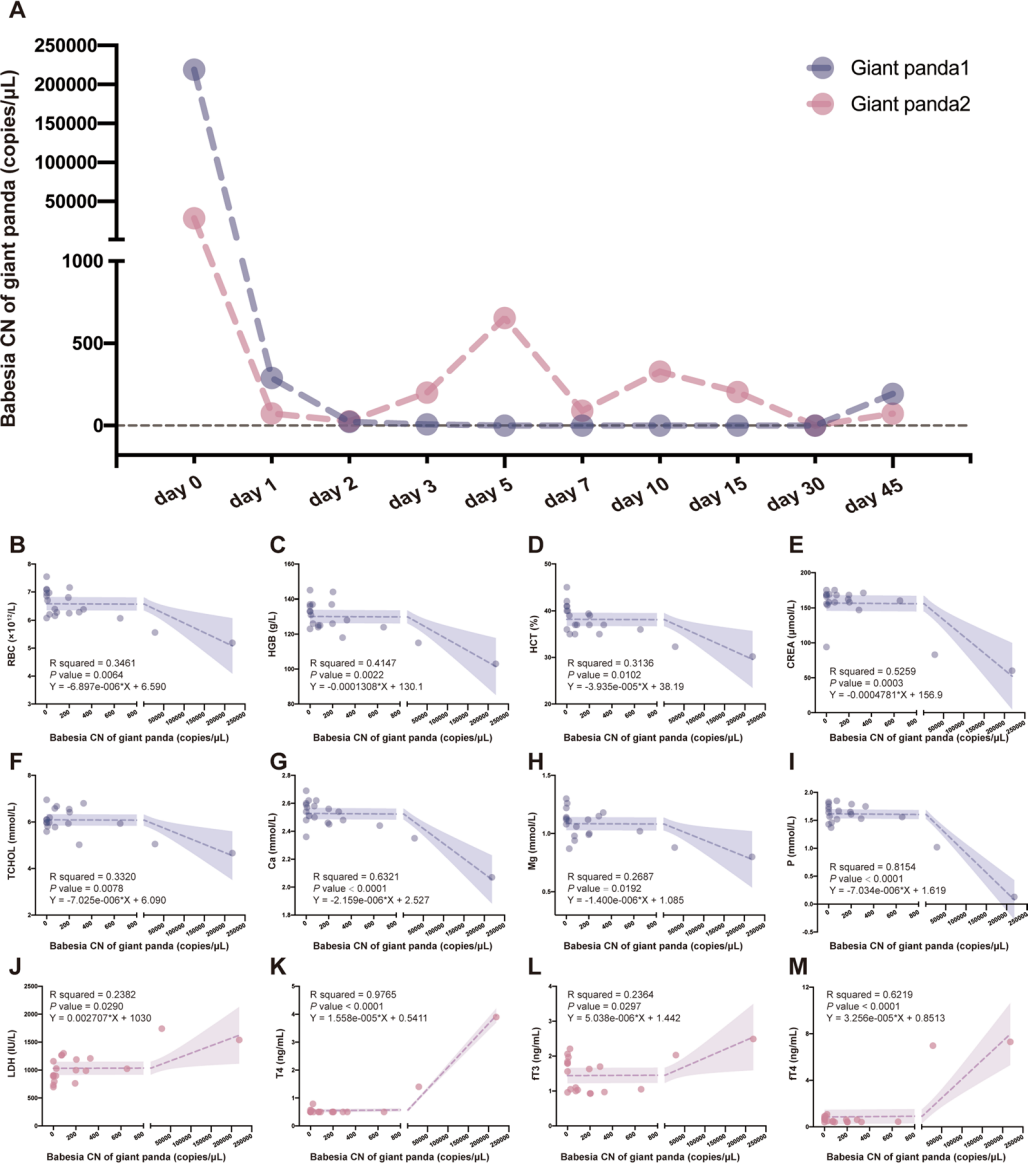
The changes in *Babesia* sp. PCR monitoring results and their correlation with blood test parameter indicators. **A** Changes in *Babesia* sp. content detected by qPCR before, during and after the combined treatment. The x-axis represents the treatment and monitoring period, and the y-axis represents the specific values of *Babesia* sp. CN (copies/μl). Red and blue indicate different subjects. The correlation results between the *Babesia* sp. PCR detection content with: **B** red blood cells (RBC); **C** hemoglobin (HGB); **D** hematocrit (HCT); **E** creatinine (CREA); **F** total cholesterol (TCHOL); **G** calcium (Ca); **H** magnesium (Mg);**I** phosphate (P); **J** lactic dehydrogenase (LDH); **K** tetraiodothyronine (T4); **L** free triiodothyronine (fT3) and (**M**) free tetraiodothyronine (fT4). The x-axis represents the specific values of *Babesia* sp. CN (copies/μl), and the y-axis represents the specific values of these indicators. Red indicates a significant positive correlation, while blue indicates a significant negative correlation

qPCR was utilized to monitor the effects of combination treatment. As shown in the Fig. 3A, the *Babesia* sp. DNA copies decreased rapidly after 1 day of treatment in both pandas. The parasites were no longer detected from the 2nd day post-treatment in giant panda 1. Moreover, parasites reappeared in the peripheral blood of giant panda 1 at 45 days post-treatment, and low *Babesia* sp. DNA copies persisted in the peripheral blood of giant panda 2 after treatment. The univariate linear regression analysis demonstrated a significant negative correlation between the *Babesia* sp. CN and RBC (*R*^2^ = 0.3461, *p* = 0.0064, Fig. 3B), HGB (*R*^2^ = 0.4147, *p* = 0.0022, Fig. 3C), HCT (*R*^2^ = 0.3136, *p* = 0.0102, Fig. 3D), CREA (*R*^2^ = 0.5259, *p* = 0.0003, Fig. 3E), TCHOL (R^2^ = 0.3320, *p* = 0.0078, Fig. 3F), Ca (R^2^ = 0.6321, *p* < 0.0001, Fig. 3G), Mg (R^2^ = 0.2687, *p* = 0.0192, Fig. 3H) and P (R^2^ = 0.8154, *p* < 0.0001, Fig. 3I). Additionally, the *Babesia* sp. CN of giant panda showed a significant positive correlation with the LDH (*R*^2^ = 0.2382, *p* = 0.029, Fig. 3J), T4 (*R*^2^ = 0.9765, *p* < 0.0001, Fig. 3K), fT3 (*R*^2^ = 0.0297, *p* = 0.0297, Fig. 3L) and fT4 (*R*^2^ = 0.5219, *p* < 0.0001, Fig. 3M). The results showed that treatment with atovaquone and azithromycin significantly reduces the presence of *Babesia* sp. in giant pandas.

## Discussion

Giant pandas are threatened by *Babesia* spp. in their natural habitats [4]. These parasites typically reside within the erythrocytes, remaining dormant when the host’s immune system functions normally [17]. At this stage, the infected giant pandas are at risk of relapse and serve as potential reservoirs for the transmission of *Babesia* spp.; any decline in immune function due to viral or bacterial infections can exacerbate the condition [18]. The clinical symptoms of babesiosis in giant pandas are similar to those observed in Bornean sun bears (*Helarctos malayanus euryspilus Horsfield*), including mild anemia and decreased platelet counts [19]. However, the clinical symptoms of babesiosis can vary in other bear species. For example, in zoo-housed polar bears (*Ursus maritimus*), babesiosis primarily causes acute loss of appetite and lethargy, with hematological examinations indicating liver and kidney failure [20]. In Formosan black bears (*Ursus thibetanus formosanus*), the *Babesia* sp. infection is mainly characterized by a significantly higher erythrocyte sedimentation rate (ESR; at 30 min and 1 h) and elevated globulin levels compared to uninfected bears [21]. Typically, rescued wild giant pandas would be released back into their original habitats. However, considering that some rescued wild pandas no longer possess the basic skills required for survival in the wild, relocation to managed captivity zoo areas is feasible. This is done to enhance the genetic diversity of the captive giant panda population [22, 23]. In natural environments, babesiosis is primarily transmitted between hosts through tick bites [21]. However, there have also been reports of tick-borne transmission of babesiosis occurring in captive settings [20]. In captivity, non-vectorial routes of transmission such as fighting and biting among giant pandas, as well as blood transfusions, can potentially transmit *Babesia* spp. Although there is no current literature supporting the transmission of *Babesia* through semen, transplacental infections were observed in dogs, resulting in stillbirths [24, 25]. The disease was transmitted by placenta not only in dogs [25] but also in humans [26] and cows [27]. Surviving offspring may suffer from hypothermia, anemia, anorexia and coma and subsequently die [28]. Therefore, this poses a risk of introducing diseases such as babesiosis to the captive giant panda population. Therefore, identifying a treatment method suitable for addressing *Babesia* spp. infections in giant pandas is critical for maintaining the health of captive giant panda populations, controlling potential babesiosis outbreaks and increasing the success rate of reintroducing captive giant pandas into the wild.

Detection methods for *Babesia* sp. primarily include blood smear microscopy, serological tests (such as indirect fluorescent antibody test [IFA] and enzyme-linked immunosorbent assay [ELISA]) and molecular biology tests (such as PCR) [29–32]. Notably, serological testing may not be suitable for all *Babesia* species and hosts, and there may be cross-reactivity among different species [33]. For instance, an ELISA assay developed using Apical Membrane Antigen 1 (AMA1) as a candidate diagnostic antigen can simultaneously diagnose six strains of *Babesia motasi* in sheep [34], whereas an ELISA assay based on recombinant merozoite surface antigen 1 (rMSA-1) has been established for diagnosing *Babesia bovis* infections in cattle [31]. Direct observation of Babesia parasites in blood smears stained with Giemsa is not only cost-effective and rapid, but also the subsequent smear of capillary blood and concentration line technique further improve the detection sensitivity, which has been widely used in the clinical detection of *Babesia* [29, 35]. Subsequent IFA and ELISA tests further enhance specificity and sensitivity; however, they may fail to detect the disease in its early stages because of the absence of seroconversion [36]. Currently, the most widely used PCR and qPCR methods accurately detect low concentrations of *Babesia* sp. DNA, proving particularly effective for early-stage infections [32]. However, when the parasite load is below the detection limit, PCR may fail to detect window period cases [36]. The qPCR then shows the advantages of high sensitivity and specificity. Notably, it can realize to quantitative detection of pathogenic concentration. It has been widely used in the quantitative detection of pathogens, such as in bacterial [37], virus [38], parasitic [39] and other biochemical markers such as ferroptosis-related genes [40]. The real-time fluorescent quantitative PCR (qPCR) used in this study can complete the entire process from blood collection to obtaining quantitative results within 3 h. It is rapid, accurate and sensitive.

Since the advent of pharmacological treatments against *Babesia microti* infection in 1982, to date, no curative treatment has been identified that can completely eradicate human babesiosis [41]. Initial treatments for canine babesiosis frequently included chloroquine, quinine, pentamidine and exchange transfusions, with splenectomy sometimes performed to improve patient survival [42]. However, these approaches for treating canine babesiosis often yielded suboptimal outcomes, had prolonged treatment durations and were associated with significant side effects and high recurrence rates [18]. In the development and utilization of pharmaceuticals in veterinary medicine, researchers have explored using imidocarb dipropionate, isothiocyanate and iiminazene aceturate to reduce the recurrence rate of human and canine babesiosis [43, 44]. However, these treatments of canine babesiosis were unsatisfactory, resulting in additional parasympathetic symptoms such as salivation, vomiting and nasal discharge as well as less common side effects including generalized weakness, fever, muscle spasms, diarrhea, renal tubular necrosis, hepatic necrosis and allergic reactions [45]. Besides, the toxic side effects experienced by the treated dogs can lead to brain, liver and kidney failure [7]. Moreover, various antibiotics are employed as monotherapies to combat *Babesia* sp. infections, such as doxycycline, clindamycin and enrofloxacin, but these often result in short-term relapse after clinical symptoms subside or the treatment concludes, failing to effectively cure the infection [9–11].

Beyond monotherapy, combination drug regimens are increasingly utilized in the treatment of babesiosis, demonstrating improved therapeutic outcomes. A study involving the treatment of 13 dogs with *B. gibsoni* infection found that a triple-drug regimen consisting of clindamycin, diminazene aceturate and imidocarb dipropionate had an 84.0% success rate yet showed a high rate of short-term relapse [46]. A combination therapy consisting of doxycycline, enrofloxacin and metronidazole achieved an 83.3% success rate over 6 weeks in treating canine babesiosis caused by *B. gibsoni* [14]. However, the inclusion of diminazene aceturate increased the treatment efficacy to 85.7% [14].

The concurrent administration of atovaquone and azithromycin, known to exhibit either additive or synergistic therapeutic effects, is currently regarded as the most effective treatment for babesiosis in cattle, gerbils (*Meriones unguiculatus*) and humans [47]. This regimen achieves a success rate > 95.0%, effectively reducing parasite levels to below PCR detection thresholds, associated with minimal side effects, albeit with a potential for relapse [47]. As an antiprotozoal medication, atovaquone could selectively inhibit the mitochondrial electron transport in protozoa, leading to the suppression of pyrimidine and adenosine triphosphate synthesis, and it has currently become the preferred agent for the treatment of babesiosis[8]. However, widespread use of atovaquone is limited because of four main factors: first, it is unregistered in many countries, making it unavailable for purchase; second, it is expensive; third, there is a tendency to relapse when it is used as monotherapy; fourth, there are frequent mutations in the cytochrome b (CYTb) gene, leading to amino acid substitutions at the atovaquone binding site, causing atovaquone resistance [48–50]. Azithromycin, a macrolide antibiotic, binds to the 50S ribosomal subunit of bacteria, thereby inhibiting the translation of messenger RNA (mRNA) and bacterial protein synthesis [12]. Azithromycin has demonstrated efficacy against *Plasmodium falciparum* and *Toxoplasma gondii*; however, direct evidence regarding its mechanism in protozoan pathogens is still lacking [12]. It is currently widely used in the treatment of *Babesia* spp. infections in dogs and mice, yet it can cause side effects including stomach discomfort, cramps, nausea and diarrhea [51, 52]. The combination of atovaquone and azithromycin allows for different mechanisms to work together on pathogens to improve therapeutic efficacy; on the other hand, the synergistic effect of atovaquone and azithromycin reduced the total dose required for each drug, thereby reducing their side effects [24, 53].

This study employed a combination of atovaquone and azithromycin for the treatment of babesiosis in giant pandas. The rationale was to ensure a high success rate while minimizing side effects and stress stimuli associated with injections. After a 10-day course of combination therapy, the levels of *Babesia* sp. in two giant pandas were reduced to below the PCR assay detection threshold, and the monitored blood parameters remained stable, with no significant clinical side effects observed, demonstrating excellent therapeutic efficacy and safety. In both human and animal cases of malaria and babesiosis, anemia is primarily caused by immune responses, followed by the damaging effects of the parasites within red blood cells [54]. Consistent with the results of this study, all individuals exhibited mild anemia prior to treatment. With the initiation of combined medication, the parasitic load significantly decreased, accompanied by an increase in red blood cells, hemoglobin and hematocrit. Parasites must extract host nutrients to support their continuous proliferation, leading to metabolically impacted host red blood cells that respond to infection by altering their metabolic profile [55]. In an effort to maintain homeostasis, this results in elevated levels of LDH [55]. The combination of metabolomics and high-resolution electron microscopy revealed that *Babesia* infection significantly alters host lipid metabolism and energy metabolism pathways [56]. This may explain the observed alterations in blood TCHOL levels in giant pandas. The elevated serum creatinine levels in giant pandas are likely primarily due to kidney insufficiency caused by babesiosis [57]. Additionally, disruptions in cellular metabolism can result in blood trace element levels falling below the normal range, leading to hypocalcemia [58]. We found a significant positive correlation between the blood parasitic CN in giant pandas and various thyroid hormones (T4, fT3 and fT4). Recent studies have demonstrated that infections by parasites such as *Entamoeba histolytica* and *Giardia lamblia* can result in elevated levels of anti-TG, anti-TPO and IgE [59]. Children with malaria exhibit significantly elevated blood levels of thyroid hormones T3, ft3 and ft4, which show a significant positive correlation with parasite load [60]. The parasitic infections may influence the hypothalamic-pituitary-thyroid axis and peripheral thyroxine metabolism, affecting thyroid function via immune response pathways [61]. Notably, several studies have found that babesiosis or malaria infections can lead to decreased thyroid function, which may be associated with increased IL-6 concentrations [62, 63]. How *Babesia* infection affects blood trace elements requires further research.

This study evaluated the efficacy and safety of using atovaquone and azithromycin in combination to treat *Babesia* infections in giant pandas. The results suggest that for treating *Babesia* infections in large wild animals such as giant pandas, the combined use of atovaquone and azithromycin rapidly improves anemia symptoms. It is effective with a short treatment duration and minimal side effects. This drug combination can be recommended for the emergency treatment of acute *Babesia* sp. infections in giant pandas. Future research on the prevention of *Babesia* sp. infections in giant pandas can focus on the following aspects: (1) work on novel effective drugs and drug combinations, especially those derived from botanical medicines [64]; (2) develop and update rapid and user-friendly high-precision early disease-screening techniques; (3) seek specific early infection biomarkers; (4) develop effective vaccines for *Babesia* infections in giant pandas; (5) strengthen zoo management and control the activities of rodents, ticks and stray dogs in the area; (6) establish and implement comprehensive health examination procedures as well as transfer and isolation procedures for wild and captive giant pandas; (7) actively implement preventive measures, such as developing acaricides that are safe and non-toxic to host animals, establishing feasible techniques for eliminating ticks from the body surface or environment and incorporating tick inspections into routine health examinations.

## Conclusions

This study demonstrates that the combination of atovaquone and azithromycin is highly effective in treating chronic *Babesia* sp. infections in giant pandas. The drug combination can rapidly eradicate parasitemia to levels below the detectable threshold of qPCR testing without causing adverse reactions. However, there is a possibility of recurrence after 45 days. The combined use of atovaquone and azithromycin is also suitable for the emergency treatment of acute *Babesia* sp. infections in giant pandas.

## Supplementary Information

Additional file 1: Table S1. The hematological, biochemical and thyroid hormone parameter difference of two giant pandas between pre-treatment (day 0) and each period in treatment (day 1, day 2, day 3, day 5, day 7, day 10) and monitoring phase (day 15, day 30, day 45).

## Data Availability

No datasets were generated or analysed during the current study.
