# Supplementary material for: Efficacy of azithromycin combined with compounded atovaquone in treating babesiosis in giant pandas
Source: Parasit Vectors. 2024 Dec 23;17:531. doi: 10.1186/s13071-024-06615-9 (PMC11665231; doi:10.1186/s13071-024-06615-9)
Supplement: Supplementary file 1 — Additional file 1: Table S1. The hematological, biochemical and thyroid hormone parameter difference of two giant pandas between pre-treatment (day 0) and each period in treatment (day 1, day 2, day 3, day 5, day 7, day 10) and monitoring phase (day 15, day 30, day 45). [file 13071_2024_6615_MOESM1_ESM.doc]

**Efficacy of Azithromycin Combined with Compounded Atovaquone in Treating Babesiosis in Giant Pandas**

**Table S1. The Hematological, biochemical and thyroid hormone parameters difference of 2 giant pandas between pre-treatment (day 0) and each period in treatment (day 1, day 2, day 3, day 5, day 7, day 10) and monitoring phase (day 15, day 30, day 45).**

| Blood test | Parameter | vs. day1 | vs. day2 | vs. day3 | vs. day5 | vs. day7 | vs. day10 | vs. day15 | vs. day30 | vs. day45 |
| --- | --- | --- | --- | --- | --- | --- | --- | --- | --- | --- |
|  |  | *p* - Value | *p* - Value | *p* - Value | *p* - Value | *p* - Value | *p* - Value | *p* - Value | *p* - Value | *p* - Value |
| Hematological test | WBC | 0.180 | 0.655 | 0.655 | 0.655 | 0.655 | 0.655 | 0.655 | 0.180 | 0.180 |
|  | LYM | 0.180 | 0.655 | 0.655 | 0.655 | 0.655 | 0.180 | 0.180 | 0.655 | 0.655 |
|  | MON | 0.655 | 0.180 | 0.180 | 0.180 | 0.180 | 0.180 | 0.180 | 0.180 | 0.180 |
|  | EOS | 0.317 | 1.000 | 1.000 | 1.000 | 1.000 | 0.317 | 1.000 | 1.000 | 1.000 |
|  | BAS | 0.180 | 0.180 | 0.317 | 0.157 | 0.655 | 1.000 | 0.157 | 1.000 | 0.317 |
|  | NEU | 0.180 | 0.655 | 0.180 | 0.655 | 0.655 | 0.655 | 0.180 | 0.655 | 0.655 |
|  | RBC | 0.180 | 0.180 | 0.180 | 0.180 | 0.180 | 0.180 | 0.180 | 0.180 | 0.180 |
|  | HGB | 0.180 | 0.180 | 0.180 | 0.180 | 0.180 | 0.180 | 0.180 | 0.180 | 0.180 |
|  | HCT | 0.180 | 0.180 | 0.180 | 0.180 | 0.180 | 0.180 | 0.180 | 0.180 | 0.180 |
|  | MCV | 0.655 | 0.655 | 0.180 | 0.180 | 0.180 | 0.180 | 0.180 | 0.655 | 0.180 |
|  | MCH | 0.180 | 0.157 | 0.180 | 0.180 | 0.180 | 0.180 | 0.180 | 0.180 | 0.655 |
|  | MCHC | 0.180 | 0.655 | 0.157 | 0.180 | 0.180 | 0.180 | 1.000 | 0.180 | 0.180 |
|  | RDW-CV | 0.317 | 0.180 | 0.180 | 0.180 | 0.180 | 0.180 | 0.180 | 0.180 | 0.157 |
|  | RDW-SD | 0.655 | 0.317 | 0.180 | 0.655 | 0.180 | 0.180 | 0.180 | 0.180 | 0.180 |
|  | PLT | 0.655 | 0.180 | 0.180 | 0.180 | 0.180 | 0.180 | 0.655 | 0.180 | 0.180 |
|  | MPV | 0.180 | 0.655 | 0.655 | 0.655 | 0.655 | 0.655 | 0.180 | 0.655 | 0.180 |
|  | PCT | 0.180 | 0.180 | 0.180 | 0.157 | 0.180 | 0.180 | 0.655 | 0.180 | 0.655 |
| Biochemical test | ALT(IU/L) | 0.180 | 0.180 | 0.180 | 0.180 | 0.180 | 0.180 | 0.180 | 0.180 | 0.180 |
|  | AST(IU/L) | 0.180 | 0.655 | 0.180 | 0.655 | 0.180 | 0.180 | 0.180 | 0.180 | 0.655 |
|  | AKP (IU/L) | 0.655 | 0.180 | 0.180 | 0.655 | 0.180 | 0.180 | 0.180 | 0.180 | 0.180 |
|  | TP (g/L) | 0.180 | 0.655 | 0.180 | 0.655 | 0.180 | 0.180 | 0.180 | 0.180 | 0.180 |
|  | ALB (g/L) | 0.180 | 0.180 | 0.180 | 0.180 | 0.157 | 0.180 | 0.157 | 0.180 | 0.655 |
|  | GLB (g/L) | 0.180 | 0.180 | 0.180 | 0.655 | 0.180 | 0.180 | 0.180 | 0.180 | 0.180 |
|  | TBIL (μmol/L) | 0.180 | 0.655 | 0.655 | 0.655 | 0.655 | 0.180 | 0.180 | 0.655 | 0.180 |
|  | DBIL (μmol/L) | 0.180 | 0.180 | 0.180 | 0.180 | 0.655 | 0.655 | 0.655 | 0.655 | 0.180 |
|  | IBIL (μmol/L) | 0.317 | 0.655 | 0.655 | 0.655 | 0.317 | 0.180 | 0.655 | 0.180 | 0.317 |
|  | GLU(mmol/L) | 0.180 | 0.180 | 0.655 | 0.655 | 0.655 | 0.180 | 0.180 | 0.655 | 0.180 |
|  | BUN (mmol/L) | 0.655 | 0.180 | 0.180 | 0.180 | 0.180 | 0.180 | 0.180 | 0.180 | 0.180 |
|  | CREA (μmol/L) | 0.180 | 0.180 | 0.180 | 0.180 | 0.180 | 0.180 | 0.180 | 0.180 | 0.180 |
|  | TRIG (mmol/L) | 0.180 | 0.180 | 0.317 | 0.655 | 0.655 | 0.180 | 0.180 | 0.180 | 0.180 |
|  | TCHOL (mmol/L) | 0.180 | 0.180 | 0.180 | 0.180 | 0.180 | 0.180 | 0.180 | 0.180 | 0.180 |
|  | HDL-C (mmol/L) | 0.180 | 0.180 | 0.180 | 0.180 | 0.180 | 0.180 | 0.180 | 0.180 | 0.180 |
|  | LDL-C (mmol/L) | 0.180 | 0.180 | 0.180 | 0.180 | 0.180 | 0.180 | 0.655 | 0.655 | 0.655 |
|  | LDH(IU/L) | 0.180 | 0.180 | 0.180 | 0.180 | 0.180 | 0.180 | 0.180 | 0.180 | 0.180 |
|  | Ca(mmol/L) | 0.180 | 0.180 | 0.180 | 0.180 | 0.180 | 0.180 | 0.180 | 0.180 | 0.180 |
|  | Mg(mmol/L) | 0.180 | 0.655 | 0.180 | 0.180 | 0.180 | 0.180 | 0.180 | 0.180 | 0.180 |
|  | P(mmol/L) | 0.180 | 0.180 | 0.180 | 0.180 | 0.180 | 0.180 | 0.180 | 0.180 | 0.180 |
|  | CO2(mmol/L) | 0.180 | 0.317 | 0.180 | 0.655 | 0.655 | 0.317 | 0.317 | 0.317 | 0.180 |
|  | AMY(IU/L) | 0.180 | 0.655 | 0.180 | 0.180 | 0.180 | 0.180 | 0.180 | 0.180 | 0.180 |
| Thyroid hormone test | T3 (nmol/L) | 0.655 | 0.655 | 0.655 | 0.655 | 0.655 | 0.655 | 0.655 | 0.317 | 0.655 |
|  | T4 (nmol/L) | 0.180 | 0.180 | 0.180 | 0.180 | 0.180 | 0.180 | 0.180 | 0.180 | 0.180 |
|  | fT3 (pmol/L) | 0.180 | 0.180 | 0.180 | 0.180 | 0.180 | 0.180 | 0.180 | 0.180 | 0.180 |
|  | fT4 (pmol/L) | 0.180 | 0.655 | 0.655 | 0.655 | 0.655 | 0.180 | 0.180 | 0.180 | 0.180 |

Significance versus pretreatment day 0: * *p* < 0.05; ** *p* <0.01; *** *p* < 0.001.
